# Impact of Uncontrolled Diabetes on Myocardial Global Longitudinal Strain: A Case–Control Study

**DOI:** 10.31083/RCM38967

**Published:** 2025-06-27

**Authors:** Khaled Elenizi, Rasha Alharthi, Abdullah Alanazi, Nasser Alotaibi, Mubarak Alajmi, Abdulrahman Alsubaie, Sahar Gamil, Mohammed Alqahtani

**Affiliations:** ^1^Department of Internal Medicine, College of Medicine, Prince Sattam bin Abdulaziz University, 11942 Alkharj, Saudi Arabia; ^2^Department of Cardiology, Dr. Sulaiman Al-Habib Hospital, 11635 Riyadh, Saudi Arabia; ^3^Department of Clinical Pharmacy, College of Pharmacy, Jouf University, 72388 Sakaka, Saudi Arabia; ^4^General Internal Medicine Division, Department of Medicine, King Saud University Medical City, King Saud University, 11451 Riyadh, Saudi Arabia; ^5^Department of Basic Medical Sciences, College of Medicine, Prince Sattam Bin Abdulaziz University, 11942 Al-Kharj, Saudi Arabia

**Keywords:** diabetes mellitus, three-dimensional speckle-tracking, echocardiography, LV function, myocardial strain

## Abstract

**Background:**

Subclinical systolic dysfunction due to diabetic microangiopathy and its impact on left ventricular (LV) function remains unclear. Myocardial deformation (strain) imaging can detect LV systolic dysfunction earlier than conventional ejection fraction evaluations. Thus, this study aimed to examine the relationship between uncontrolled diabetes and impaired LV global longitudinal strain (GLS) in patients with diabetes mellitus (DM) compared to non-diabetic individuals.

**Methods:**

A total of 76 asymptomatic patients with uncontrolled type 2 DM and 76 age- and gender-matched healthy controls underwent transthoracic echocardiography imaging. Patients with coronary artery disease, an LV ejection fraction <55%, atrial fibrillation, or inadequate echocardiographic quality were excluded. The presence of proliferative retinopathy, microalbuminuria, nephropathy, or peripheral neuropathy defines diabetic microvascular complications.

**Results:**

The absolute GLS% was significantly lower in the uncontrolled diabetic group (–18.4 ± 1.7) compared to controls (–22 ± 1.9, *p* < 0.001). Diabetic patients with complications had lower absolute GLS% values of –18.9 ± 1.7 for no complications, –17.5 ± 1.3 for one complication, and –16.8 ± 1.3 for two or more complications (*p*-value = 0.001). Regression analysis showed a positive association between complications and lower absolute GLS% (β = 0.41, *p* < 0.001). No significant difference was found in LV mass between hypertensive (155.1 ± 40.4) and non-hypertensive individuals (139.8 ± 44.3; *p*-value = 0.19).

**Conclusion:**

Uncontrolled diabetes and the presence of complications were associated with lower absolute GLS% values, suggesting impaired myocardial deformation. These findings highlight the importance of monitoring GLS% as a potential marker for cardiac involvement in diabetic patients.

## 1. Introduction

Diabetes mellitus (DM) has long been recognized as a major risk factor for cardiovascular disease, contributing to the development of both macrovascular and microvascular complications and the development of heart failure [1, 2]. Among the most significant cardiovascular consequences of diabetes is diabetic cardiomyopathy, a condition characterized by structural and functional alterations of the myocardium independent of coronary artery disease, valvular disease, or hypertension [3, 4].

Left ventricular (LV) systolic dysfunction, a hallmark of diabetic cardiomyopathy, often presents subclinically and progresses over time, ultimately contributing to heart failure and increased mortality [5]. Left ventricular ejection fraction (LVEF), the most widely used parameter for evaluation of systolic function, has low sensitivity for the assessment of early dysfunction in myocardial contractility [6]. Emerging evidence supports the use of echocardiographic strain analysis to detect early subclinical LV dysfunction in diabetic patients, even with preserved ejection fraction [7]. The impact of glycemic control on these early changes remains under investigation. Poor glycemic control was linked to impaired global longitudinal strain (GLS) and other systolic strain measures, suggesting early myocardial damage [8]. Conversely, a study suggested that intensive glycemic control might reverse these alterations, emphasizing the importance of early and targeted intervention [9].

To our knowledge, this is the first study in a Middle Eastern population to assess the impact of suboptimally controlled diabetes on GLS using three-dimensional speckle-tracking echocardiography, with a focus on microvascular complication severity.

This study aimed to explore the relationship between glycemic control and LV systolic function in diabetic patients, using advanced echocardiographic techniques to assess subclinical LV dysfunction. By comparing poorly controlled diabetic patients to a control group, as well as those with and without diabetic complications, we sought to provide a comprehensive analysis of how glucose regulation impacts cardiac health. Additionally, this study highlighted the potential for early detection of myocardial dysfunction, offering insights into therapeutic strategies to reduce cardiovascular risk in diabetic populations.

## 2. Materials and Methods

### 2.1 Study Population, Setting, and Eligibility Criteria

This study included 76 outpatients with uncontrolled type 2 diabetes from Prince Sattam University Hospital in Al-Kharj, Saudi Arabia, from June to August 2024, diagnosed according to the 2024 American Diabetes Association guidelines, with a LVEF of ≥55%. Exclusion criteria included coronary artery disease, atrial fibrillation, and poor echocardiographic quality. Hypertension status was documented, and only controlled hypertension patients were included. Controlled hypertension was defined as blood pressure <130/80 mmHg (per 2024 American College of Cardiology/American Heart Association guidelines) on two measurements taken two minutes apart after a 10-minute supine rest, with patients on stable antihypertensive therapy for at least three months.

A control group of 76 individuals with age- and gender-matched hospital patients without diabetes (defined by normal glycated hemoglobin (HbA1c), postprandial glucose, and fasting glucose levels), hypertension, or coronary artery disease, and with normal electrocardiogram (ECG) and echocardiographic findings, were selected concurrently.

### 2.2 Sample Size Calculation

The sample size (n = 76 per group) was calculated to detect a 2% GLS% difference (SD = 1.9%), with 80% power and α = 0.05, requiring at least 72 participants per group.

### 2.3 Data Collection

Data included demographics (age, sex), anthropometric measurements (height, weight, body mass index (BMI)), blood pressure readings, hypertension status, metabolic indicators (HbA1c, glucose, diabetic complications), smoking status, dyslipidemia, hemoglobin, creatinine, and the presence of ischemic heart disease, atrial fibrillation, and heart failure symptoms. Medication usage, including various antidiabetic (e.g., metformin, insulin) and cardiovascular drugs (e.g., angiotensin-converting enzyme (ACE) inhibitors, beta-blockers), will be recorded. Patients with known coronary artery disease (CAD), angina, wall motion abnormalities, or ischemic ECG changes were excluded. Routine stress testing or coronary imaging was not performed due to the asymptomatic status of the cohort and preserved LVEF, though this limits the ability to exclude subclinical CAD.

### 2.4 Outcome Measures

Blood pressure and heart rate were measured using an automated digital oscillometric sphygmomanometer after a 10-minute supine rest in a controlled environment (23 °C). Two measurements were taken two minutes apart, and the mean value was used for analysis.

**Fig. 1.**
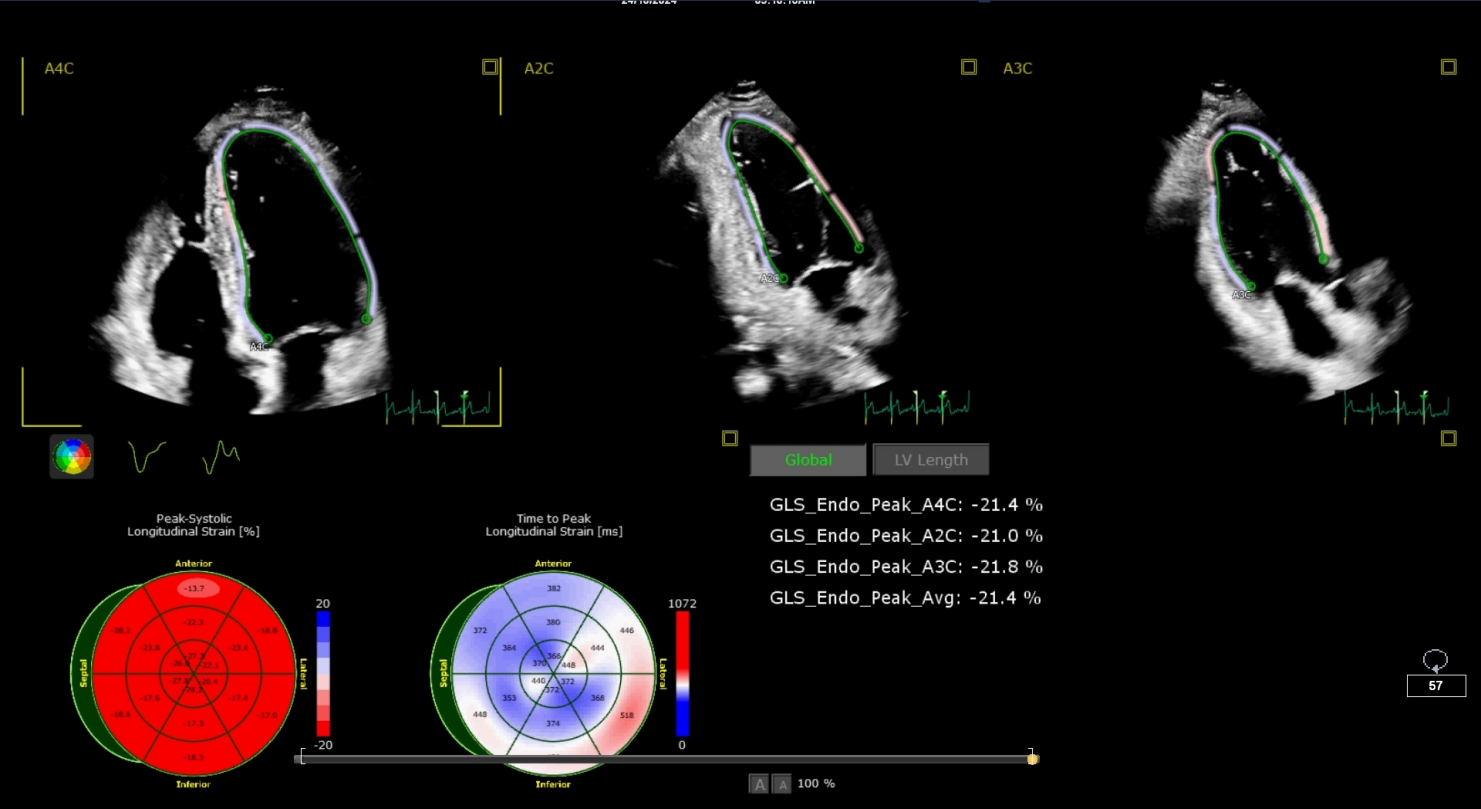
**Illustration of global longitudinal strain (GLS)% for a normal patient from the control group**.

All participants undergone transthoracic echocardiography with a Philips EPIQ CVxi system. Measurements were included for left atrial volume (Simpson’s method), LV dimensions, wall thickness, and LVEF. Diastolic function was assessed using the E/A ratio and tissue Doppler imaging (E and A velocities). Additional parameters included LV mass, volumes, tricuspid regurgitation velocity, and GLS% (Fig. 1).

### 2.5 Reliability of the Data Collected

To ensure measurement consistency, echocardiograms for 10 random subjects were repeated by the same sonographer after seven days. Intra-observer variability was assessed by having the same observer analyze the data twice, a week apart, while interobserver variability was evaluated by a second observer independently analyzing the same data. Reproducibility was quantified as the mean percent error. Intraobserver and interobserver reproducibility of transthoracic echocardiography measurement was assessed by repeating the measurement of GLS% for 10 random individuals. Intraobserver reproducibility was assessed by measuring the interclass coefficient (ICC) along with its 95% confidence intervals (CIs) of 2 readings by the same observer, 1 week apart. Interobserver reproducibility was assessed by measuring the ICC and its 95% CI for readings by 2 different observers. Observers were blinded to previous measurements. ICC ≥0.75 was considered as good, 0.4 < ICC < 0.75 as moderate and ICC ≤0.4 as poor.

### 2.6 Statistical Analysis 

Statistical analysis was conducted using the Statistical Package for Social Sciences (SPSS, version 20, IBM Corporation, Chicago, IL, USA). Continuous data were expressed as means ± standard deviation, and categorical data as numbers and percentages. Variables were compared between the uncontrolled diabetics and control group using the independent Student’s *t*-test for continuous data or the chi-square test for categorical data. Correlation of different variables with GLS% was assessed using Pearson’s correlation, independent student’s *t*-test, or the one-way ANOVA test. A linear multivariate regression (enter model) analysis was performed to look for the independent factors affecting the GLS% in the uncontrolled diabetic groups, *p*-value of less than 0.05 was considered to be statistically significant.

Intraobserver and interobserver reproducibility of transthoracic echocardiography measurement was assessed by repeating the measurement of GLS% for 10 random individuals. Intraobserver reproducibility was assessed by measuring the ICC along with its 95% CI of 2 readings by the same observer, 1 week apart. Interobserver reproducibility was assessed by measuring the ICC and its 95% CI for readings by 2 different observers. Observers were blinded to previous measurements. ICC ≥0.75 was considered as good, 0.4 < ICC < 0.75 as moderate and ICC ≤0.4 as poor.

## 3. Results

A total of 135 patients with uncontrolled diabetes were consecutively enrolled from the diabetes clinic at Prince Sattam University Hospital between June and August 2024. The primary objective of the study was to evaluate GLS. Out of 135 patients assessed for eligibility, 59 were excluded for various reasons. This included 28 patients who refused to participate, 2 patients with *de novo* heart failure, 7 patients with aortic valve calcification, 2 patients with wall motion abnormalities, 2 patients with LV hypertrophy, and 6 patients with rheumatic valvular heart disease. Additionally, 5 patients were lost to follow-up, 5 had known CAD, and 2 were excluded due to inadequate image quality. Consequently, the final sample for analysis comprised 76 patients.

The mean age was 48.9 years (±13.1) in the uncontrolled diabetes group and 46.9 years (±12.8) in controls (*p*-value = 0.33). Gender distribution was (46.1% vs. 42.1% male, *p*-value = 0.62). Hypertension affected 46.1% of the uncontrolled diabetes group, with 2.6% reporting smoking. BMI was significantly higher in the uncontrolled diabetes group (31.9 kg/m^2^ ± 6.8) compared to controls (25.5 kg/m^2^ ± 5.3) (*p* < 0.001). Systolic and diastolic blood pressures were not elevated in both groups (130.9 ± 14.5 vs. 118.9 ± 8.9 mmHg, *p* < 0.001; 78.5 ± 9.2 vs. 75.4 ± 8.4 mmHg, *p*-value = 0.03). Dyslipidemia was more common in the uncontrolled diabetes group (68.4% vs. 27.4%, *p* < 0.001). Hemoglobin was similar (13.7 ± 1.6 vs. 13.4 ± 1.1, *p*-value = 0.29), and creatinine was (68.6 ± 20.6 vs. 60.8 ± 10.5, *p*-value = 0.004). The majority (69.7%) of patients reported no complications. Specific complications in the uncontrolled diabetes group included peripheral neuropathy in 14.5%, majorly (Table 1).

**Table 1.** **Patient characteristics and clinical parameters**.

Variable		Uncontrolled diabetes (N = 76)	Control (N = 76)	*p*-value (χ^2^)
Age (years)		48.9 ± 13.1	46.9 ± 12.8	0.33*
Gender				
	Male	35 (46.1%)	32 (42.1%)	0.62** (0.24)
	Female	41 (53.9%)	44 (57.9%)	
Hypertension		35 (46.1%)	—	—
Smoking		2 (2.6%)	—	—
HbA1c (%)		8.7 ± 1.2	—	—
Glucose (mmol/L)		9.8 ± 4	—	—
BMI (kg/m^2^)		31.9 ± 6.8	25.5 ± 5.3	<0.001*
Systolic BP (mmHg)		130.9 ± 14.5	118.9 ± 8.9	<0.001*
Diastolic BP (mmHg)		78.5 ± 9.2	75.4 ± 8.4	0.03*
Dyslipidemia		52/76 (68.4%)	17/62 (27.4%)	<0.001** (22.9)
Hemoglobin (g/dL)		13.7 ± 1.6	13.4 ± 1.1	0.29*
Creatinine (µmol/L)		68.6 ± 20.6	60.8 ± 10.5	0.004*
Diabetic complications				
	No complication	53 (69.7%)	—	—
	One complication	18 (23.7%)	—	—
	Two complications	5 (6.6%)	—	—
Peripheral neuropathy		11 (14.5%)	—	—
Retinopathy		10 (13.2%)	—	—
Nephropathy		7 (9.2%)	—	—

*p-value was calculated using the independent Student’s *t*-test. **p-value was calculated using the chi-square test. HbA1c, glycated hemoglobin; BMI, body mass index; BP, blood pressure.

Metformin was prescribed to 85.5% of the uncontrolled diabetes group, followed by sodium-glucose co-transporter 2 inhibitors (SGLT-2i) (53.9%) and insulin (48.7%) (Table 2).

**Table 2.** **Medication uses among the included participants**.

Medication		Uncontrolled diabetes (N = 76)	Control (N = 76)	*p*-value (χ^2^)
Diabetic treatment				
	Metformin	65 (85.5%)	—	
	DPP4-I (Gliptins)	33 (43.4%)	—	
	Insulin	37 (48.7%)	—	
	Sulfonylurea	36 (47.4%)	—	
	SGLT-2i	41 (53.9%)	—	
	Glitazones	1 (1.3%)	—	
	GLP1-RA (Trulicity)	10 (13.2%)	—	
Hypertension treatment				
	ACEI/ARB	35 (46.1%)	—	
	Beta-blockers	10 (13.2%)	—	
	Diuretics	6 (7.9%)	—	
	Calcium channel blockers	16 (21.1%)	—	
	Aspirin	16 (21.1%)	—	
	Statin	49 (64.5%)	17 (22.4%)	0.001** (27.4)

**p-value was calculated using the chi-square test. DPP4-I, dipeptidyl peptidase-4 inhibitor; SGLT-2i, sodium-glucose co-transporter 2 inhibitors; GLP1-RA, glucagon-like peptide-1 receptor agonists; ACEI/ARB, angiotensin-converting enzyme inhibitors/angiotensin receptor blockers.

Echocardiographic analysis revealed no significant differences in left ventricular end-diastolic diameter (LVEDD: 4.6 cm vs. 4.5 cm, *p*-value = 0.07) or left ventricular end-systolic diameter (LVESD: 2.8 cm vs. 2.8 cm, *p*-value = 0.15). Interventricular septal thickness in diastole (IVSTd) and left ventricular posterior wall thickness in diastole (LVPWTd) were greater in the uncontrolled group (0.91 cm vs. 0.74 cm, *p* < 0.001; 0.8 cm vs. 0.67 cm, *p* < 0.001). Both left ventricular mass (LVM) and left ventricular mass index (LVMI) were elevated (142.3 g vs. 104.9 g, *p* < 0.001; 74.9 g/m^2^ vs. 60.8 g/m^2^, *p* < 0.001). Left ventricular end-diastolic volume (LVEDV) was reduced (75.6 mL vs. 90.8 mL, *p* < 0.001), as was the left ventricular ejection fraction (LVEF: 61.9% vs. 65.2%, *p* < 0.001). Differences were observed in mitral inflow velocities, with lower mitral E (0.83 cm/s vs. 0.91 cm/s, *p*-value = 0.007) and higher mitral A (0.81 cm/s vs. 0.66 cm/s, *p* < 0.001). Septal and lateral e’ velocities were decreased (8.8 ± 2.6 vs. 11.9 ± 2.7 cm/s, *p* < 0.001; 12.5 ± 3.7 vs. 16.8 ± 3.9 cm/s, *p* < 0.001), alongside an increased lateral E/e’ ratio (7 vs. 5.5, *p* < 0.001). GLS% absolute value was significantly reduced (–18.4% vs. –22.0%, *p* < 0.001) in the uncontrolled diabetic group (Table 3).

**Table 3.** **Comparison of echocardiographic findings between uncontrolled diabetes and control groups**.

Echocardiographic parameter		Uncontrolled diabetes (N = 76)	Control (N = 76)	*p*-value (χ^2^)
Left ventricular end-diastolic diameter (LVEDD, cm)		4.6 ± 0.5	4.5 ± 0.4	0.07*
Left ventricular end-systolic diameter (LVESD, cm)		2.8 ± 0.4	2.8 ± 0.3	0.15*
Interventricular septal thickness at end-diastole (IVSTD, cm)		0.91 ± 0.18	0.74 ± 0.12	<0.001*
Left ventricular posterior wall thickness at end-diastole (LVPWTD, cm)		0.8 ± 0.15	0.67 ± 0.1	<0.001*
Left ventricular mass (LVM, g)		142.3 ± 43.2	104.9 ± 28.1	<0.001*
Left ventricular mass index (LVMI, g/m^2^)		74.9 ± 19.4	60.8 ± 12.1	<0.001*
Left ventricular end-diastolic volume (LVEDV, mL)		75.6 ± 25.2	90.8 ± 21.3	<0.001*
Left ventricular end-systolic volume (LVESV, mL)		27.8 ± 11.5	29.5 ± 9.2	0.29*
Left atrial volume (LAV, mL)		13.9 ± 4.4	14.3 ± 3.2	0.64*
Left ventricular ejection fraction (LVEF, %)		61.9 ± 2.7	65.2 ± 3.9	<0.001
Mitral peak early diastolic velocity (E, cm/s)		0.83 ± 0.17	0.91 ± 0.17	0.007*
Mitral peak late diastolic velocity (A, cm/s)		0.81 ± 0.2	0.66 ± 0.12	<0.001*
E/A ratio		1.2 ± 0.9	1.4 ± 0.2	0.09*
Mitral deceleration time (DT, ms)		210.4 ± 42.6	199.2 ± 31.4	0.06*
Septal e’ (cm/s)		8.8 ± 2.6	11.9 ± 2.7	<0.001*
Lateral e’ (cm/s)		12.5 ± 3.7	16.8 ± 3.9	<0.001*
Lateral E/e’ ratio		7 ± 2.4	5.5 ± 1.7	<0.001*
Septal E/e’ ratio		8.9 ± 3.1	7.7 ± 2.4	0.008*
Tricuspid regurgitation (TR) velocity (m/s)		1.6 ± 1.6	2.3 ± 0.5	0.014*
Global longitudinal strain (GLS, %)		–18.4 ± 1.7	–22 ± 1.9	<0.001*
Mitral inflow patterns				
	Normal	34 (44.7%)	73 (96.1%)	0.001** (48.2)
	Diastolic dysfunction (impaired relaxation)	35 (46.1%)	3 (3.9%)	
	Pseudo normalization	7 (9.2%)	0 (0%)	

*p-value was calculated using the independent Student’s *t*-test. **p-value was calculated using the chi-square test.

**Fig. 2.**
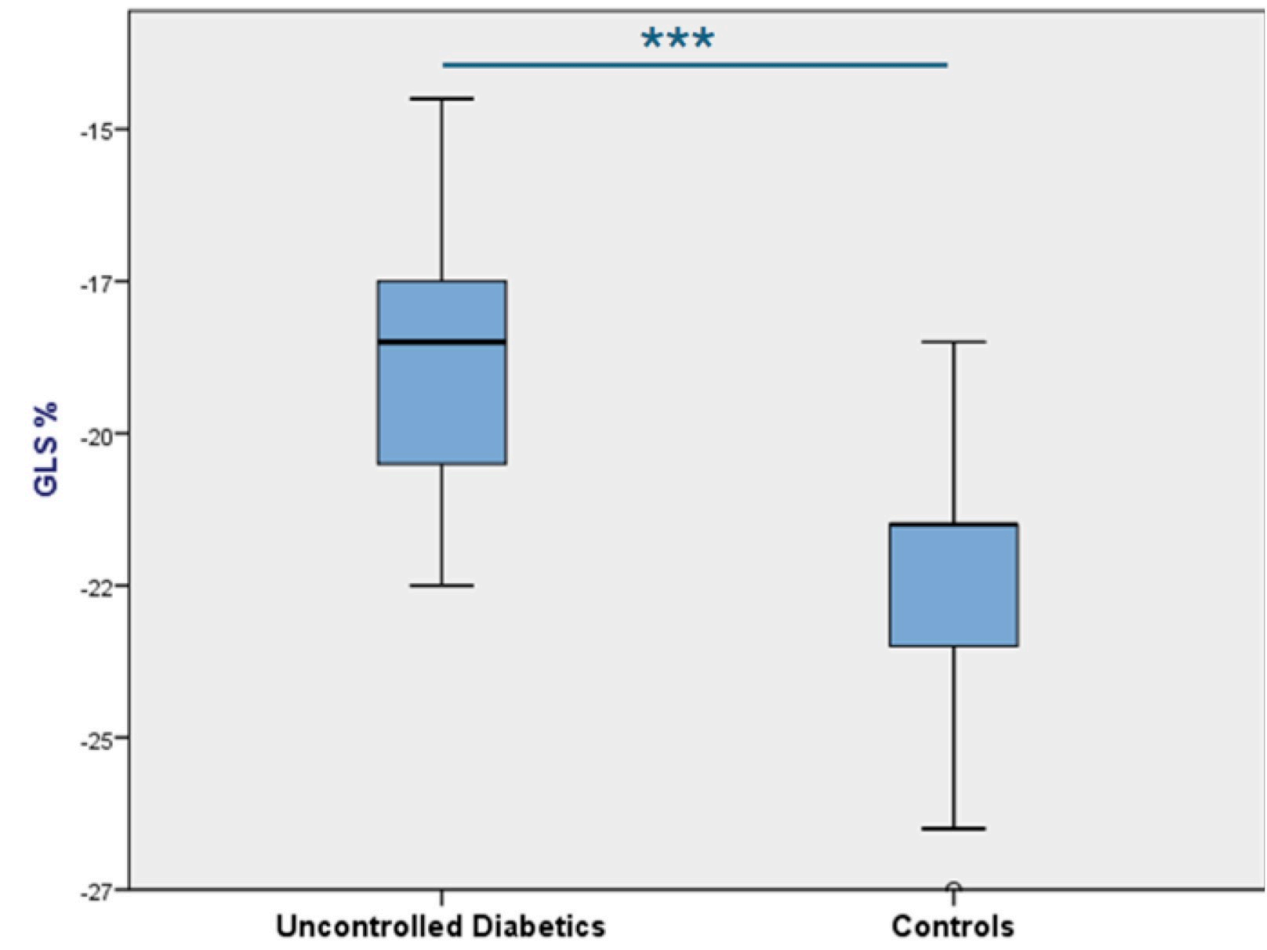
**Comparison between uncontrolled diabetes and controls GLS%**. ***p < 0.001, independent Student’s *t*-test.

The GLS% absolute value was significantly lower in the uncontrolled diabetic group (–18.4 ± 1.7) than in controls (–22 ± 1.9), with *p* < 0.001, indicating poorer myocardial deformation in uncontrolled diabetics. The boxplot highlighted this difference, with a narrower range in controls, suggesting consistent myocardial strain, while the broader range in uncontrolled diabetics reflected variability in cardiac impairment (Fig. 2).

**Fig. 3.**
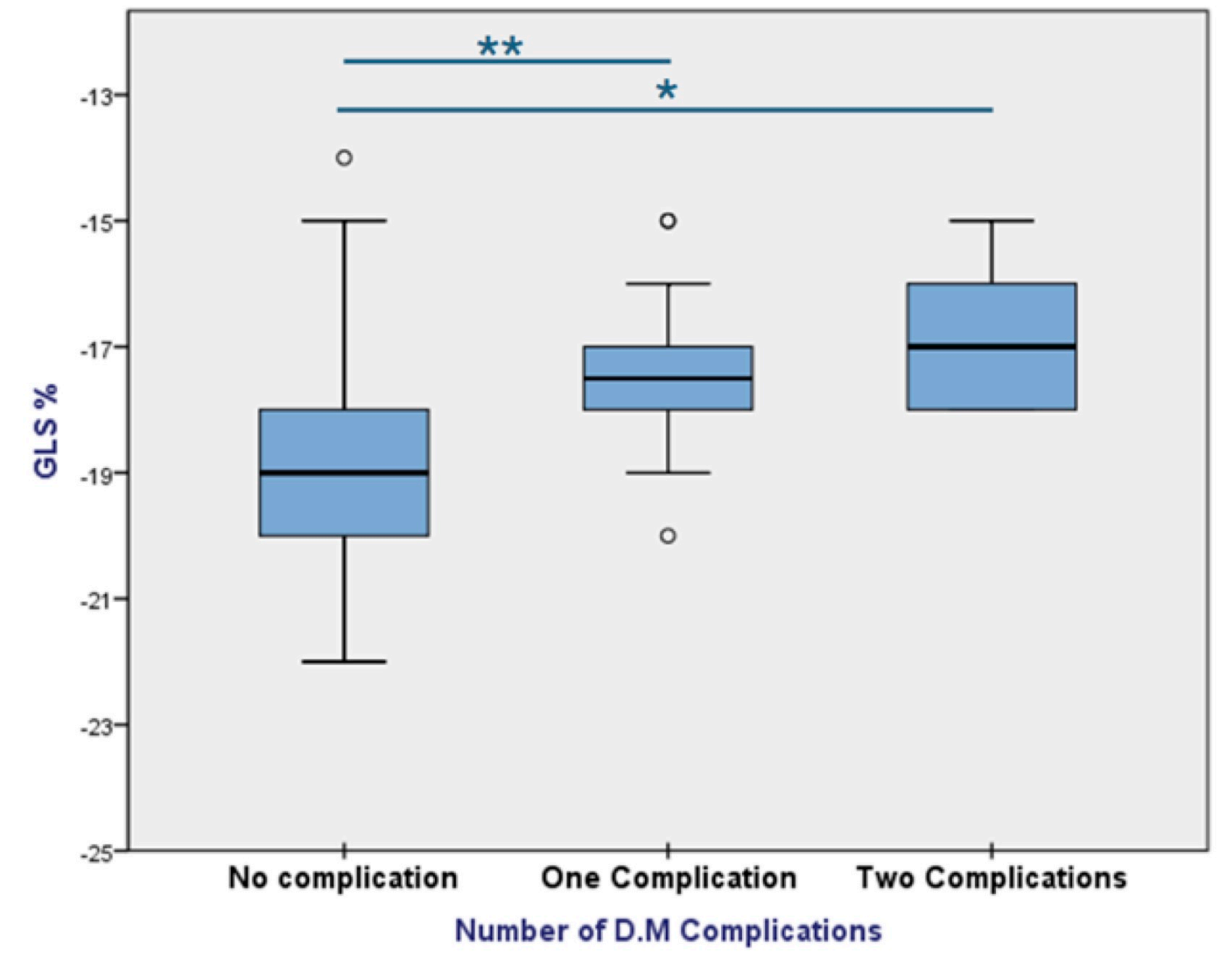
**GLS% in uncontrolled DM group according to number of complications**. *p-value < 0.05, **p-value < 0.001. DM, diabetes mellitus.

In patients with uncontrolled diabetes, those with complications had significantly lower absolute GLS% values Specifically, the GLS% was –18.9 ± 1.7 for patients with no complications, –17.5 ± 1.3 for those with one complication, and –16.8 ± 1.3 for those with two or more complications (*p*-value = 0.001). Regression analysis further showed a significant association between the presence of complications and lower absolute GLS% values (β = 0.41, *p* < 0.001), supporting the observation that diabetic complications were linked to poorer myocardial deformation (Fig. 3).

In patients with uncontrolled diabetes, GLS% did not show a significant correlation with age (correlation coefficient, r = –0.3, *p*-value = 0.77), BMI (r = –0.18, *p*-value = 0.13), systolic blood pressure (r = –0.03, *p*-value = 0.82), diastolic blood pressure (r = –0.02, *p*-value = 0.85), HbA1c levels (r = 0.001, *p*-value = 0.99), glucose levels (r = 0.05, *p*-value = 0.66), hemoglobin (r = 0.17, *p*-value = 0.14), creatinine (r = 0.16, *p*-value = 0.16), or dyslipidemia status (*p*-value = 0.76). Additionally, there was no significant difference in GLS% between groups based on sex (*p*-value = 0.09), presence of hypertension (*p*-value = 0.37), or use of specific medications (*p* > 0.1). In patients with uncontrolled diabetes, GLS% showed no significant correlation with most echocardiographic parameters, except for significant positive correlations with LVESD (r = 0.3, *p*-value = 0.007) and LV end-systolic volume (LVESV) (r = 0.25, *p*-value = 0.03).

Multiple linear regression analysis indicated that the presence of diabetic complications (β = 0.41, *p*-value < 0.001) and LVESD (β = 0.28, *p*-value = 0.03) were the most significant factors impacting GLS percentage (R = 0.26, *p* < 0.001). Both diabetic complications and an increased LVESD were associated with lower absolute GLS percentages (Table 4).

**Table 4.** **Independent factors associated with GLS% in the uncontrolled diabetic group (linear regression, enter model)**.

Variable	Beta (β)	*p*-value
Age	–0.05	0.65
Sex (male)	–0.02	0.89
BMI	–0.23	0.45
HbA1c	0.05	0.65
Presence of hypertension	0.08	0.49
Presence of diabetic complications	0.41	<0.001
LV end-systolic diameter (LVESD)	0.28	0.03
LV end-systolic volume (LVESV)	0.14	0.24

There was no significant difference between the hypertensive and non- hypertensive groups, with LV mass of 155.1 ± 40.4 in those with hypertension and 139.8 ± 44.3 in those without (*p*-value = 0.19). This suggested that hypertension does not significantly affect LV mass in uncontrolled diabetics.

Further, the intraobserver ICC for GLS% was 0.99 (95% CI: 0.97–0.99), indicating excellent consistency, while the interobserver ICC was 0.87 (95% CI: 0.6–0.96), reflecting good reliability. These findings confirmed the high reproducibility of GLS% measurements for assessing myocardial function.

## 4. Discussion

The current study found that absolute GLS% was significantly reduced in uncontrolled diabetics compared to non-diabetics (–18.4% vs. –22.0%, *p* < 0.001). Supporting this, another study reported a marked decrease in GLS% absolute values in diabetics versus controls (–12.0 ± 3.0% vs. –16.2 ± 1.9%, *p* < 0.001), with GLS% strongly correlated to diabetic microvascular complications, including cardiac autonomic neuropathy (coefficient of variation of R-R intervals (CVRR), r = 0.58, *p* < 0.001), retinopathy, and nephropathy [10].

The current study findings also highlighted an association of worsening absolute GLS% values and the severity of diabetic complications (*p* < 0.001). This underscores the link between microvascular complications and subclinical LV dysfunction, emphasizing early cardiac assessment in diabetics. Among the current sample, a total of 23 (30.3%) patients reported complications, these complications were strongly associated with the decreased absolute GLS% values. This association was also mentioned in prior studies. For example, a study by Pararajasingam *et al*. [11] showed the association between reduced absolute GLS% values and diabetic microvascular complications, where one hundred and eleven (50%) of patients had microvascular complications which showed lower levels of absolute GLS% values as the severity of complications increased.

Additionally, Chen *et al*. [12] confirmed this association as microvascular complications were significantly associated with reduced absolute GLS% values (with an odds ratio of 2.31 and *p*-value = 0.02). Another study by Zhang *et al*. [13] showed that poor glycemic control (HbA1c ≥7%) led to significant absolute GLS% reductions (–16.2 ± 2.4%) compared to controlled diabetes (–17.7 ± 2.6%, *p* < 0.05) and healthy controls (–19.1 ± 3.4%, *p* < 0.001), with HbA1c as an independent predictor of reduced absolute GLS% (β = –0.274, *p*-value = 0.024). However, in the current study, HbA1c levels (r = 0.001, *p*-value = 0.99) and glucose levels (r = 0.51, *p*-value = 0.66) showed no significant influence on GLS.

Approximately one-third of patients with uncontrolled diabetes presented with diabetes-related complications, which is consistent with findings from the previous study [13]. These complications, primarily impacting the microvasculature, were significantly associated with a decline in absolute GLS, (with a regression coefficient of β = 0.41, *p* < 0.001). This relationship underscores the principle that diabetes predominantly affects the microvasculature. Dyslipidemia was notably more prevalent in the uncontrolled diabetes group (68.4% vs. 27.4%, *p* < 0.001), though it did not show a significant association with worse GLS%. In the current study population, hypertension was not a factor influencing LV mass in uncontrolled diabetics (155.1 ± 40.4 with hypertension, 139.8 ± 44.3without hypertension, *p*-value = 0.19).

An animal study showed that diabetic rabbits experienced a progressive decline in LV absolute GLS% over nine months, with GLS% dropping to –14.56 ± 2.44% compared to –21.56 ± 2.47% (*p*-value = 0.001). The study suggested that diabetic cardiomyopathy gradually impacts myocardial layers, with the endocardium being the most vulnerable [14].

While current study focused on the general population, previous research identified that women with gestational diabetes mellitus (GDM) had lower LV absolute GLS% compared to controls (–19.3% vs. –20.1%, *p*-value = 0.002), despite no significant differences LVEF, mass, or diastolic function [15]. Another study similarly reported reduced absolute GLS% in GDM patients versus those with normal pregnancies (–17.2 ± 2.18% vs. –19.8 ± 3.34%, *p* < 0.001), with preserved LVEF [16]. Multiple regression analysis confirmed that GDM independently impacts strain values, underscoring the potential of GLS% as a monitoring tool for early cardiac changes in GDM patients.

Studies highlighted the reversibility of cardiovascular dysfunction through intensive glycemic control. One study found that six months of intensive glycemic control improved LV myocardial deformation in poorly controlled type 2 diabetes patients, with GLS% improving from –15.4 ± 3% to –18 ± 3% (*p* < 0.05) [17]. Another study showed that after 12 months, patients with type 2 diabetes who received glucagon-like peptide-1 (GLP-1) receptor agonists and SGLT-2i had strain improvements from –16 ± 4% to –18.4 ± 4.7% (*p* < 0.05) [18].

An animal study on type 2 DM rats also demonstrated that treatment with the rho-associated protein kinase (ROCK) inhibitor fasudil significantly enhanced global circumferential strain (GCS) (*p*-value = 0.003) and global circumferential strain rate (GCSR) (*p*-value = 0.021) compared to controls [19]. However, another study in well-controlled type 2 diabetes patients, who, despite achieving blood glucose management, exhibited subclinical LV systolic dysfunction, evidenced by significantly lower absolute GLS% compared to controls (–16.43 ± 2.83% vs. –18.50 ± 2.50%, *p* < 0.001) [20]. The current study, however, did not include a controlled diabetes group for comparison.

Additionally, a study revealed that DM significantly affects LV systolic function in patients with CAD, as indicated by lower peak systolic longitudinal strain (PSLS) values during rest, peak stress, and recovery phases. Specifically, global PSLS was lower in the diabetic group than in the non-diabetic group, measuring 14.5 ± 3.6% compared to 17.4 ± 4.0% at rest and 13.8 ± 3.9% versus 16.7 ± 4.0% at peak stress. DM was determined to be an independent predictor of reduced PSLS, suggesting it worsens LV dysfunction in CAD patients. Nevertheless, dobutamine stress testing did not exacerbate these differences observed at rest, indicating that coronary stenoses might mask the effects of DM during stress​ [21].

The current study results showed a significant association between lower absolute GLS values and diabetic complications, decreasing from –18.9 ± 1.7% (no complications) to –17.5 ± 1.3% (one complication) and –16.8 ± 1.3% (two or more complications, *p*-value = 0.001), suggesting GLS might be a sensitive marker for cardiac microvasculopathy in diabetes by reflecting microvascular damage that impairs myocardial function before ejection fraction changes [22]. A GLS absolute value of less than 18% might indicate cardiac microvasculopathy and a higher complication burden, though these cutoff needs validation in larger studies.

While impaired GLS in diabetes is well-documented, this study is the first to report such findings in a Saudi cohort with suboptimal glycemic control, linking GLS reduction to complications burden. Nonetheless, our sample size (n = 76 per group), though powered for the primary outcome, limits broader generalizability, and larger studies are needed to confirm these findings across diverse Middle Eastern and North African populations.

### Future Implications

Given that heart failure is a primary cause of death in diabetic patients, who are at elevated risk of developing this condition—particularly with preserved ejection fraction—early identification of myocardial damage using GLS is critical to prevent progression to symptomatic heart failure, where treatments often fail to reverse cardiac remodeling or reduce mortality. This research underscores the role of microvascular injury in the early decline of LV function, suggesting that patients with multiple microvascular issues and reduced GLS% might benefit from tailored interventions, such as intensified glycemic control or cardioprotective therapies, and consistent monitoring to slow disease progression.

## 5. Conclusion

Global longitudinal strain was significantly lower in uncontrolled diabetes patients, indicating reduced myocardial contractility. This subclinical dysfunction suggested that even in early stages, uncontrolled diabetes might impair myocardial function, underlining the importance of regular echocardiographic monitoring for early intervention.

## Availability of Data and Materials

The datasets used and analyzed during the current study are available from the corresponding author upon reasonable request.

## Supplementary Material

Supplementary material associated with this article can be found, in the online version, at https://doi.org/10.31083/RCM38967.
