# Supplementary material for: Impact of Uncontrolled Diabetes on Myocardial Global Longitudinal Strain: A Case–Control Study
Source: Rev Cardiovasc Med. 2025 Jun 27;26(6):38967. doi: 10.31083/RCM38967 (PMC12230847; doi:10.31083/RCM38967)
Supplement: Supplementary file 1 [file 2153-8174-26-6-38967-s1.zip › Supplementary Material.docx]

***Supplementary***

**Supplementary Table 1. Correlation of different variables with GLS in the uncontrolled diabetic group**

| **Category** | **Variable** | **GLS (%)** | **P-value** | **Interpretation** |
| --- | --- | --- | --- | --- |
| **Demographic and Clinical Factors** | Age | r = -0.3 | 0.77* | No significant correlation between age and GLS. |
|  | Sex (Male/Female) | -18.1 ± 1.9 / -18.7 ± 1.5 | 0.09** | No significant difference in GLS between genders. |
|  | BMI | r = -0.18 | 0.13* | No significant correlation between BMI and GLS. |
|  | Systolic BP | r = -0.03 | 0.82* | No significant correlation between systolic BP and GLS. |
|  | Diastolic BP | r = -0.02 | 0.85* | No significant correlation between diastolic BP and GLS. |
| **Laboratory Markers** | HbA1c | r = 0.001 | 0.99* | No significant correlation between HbA1c levels and GLS. |
|  | Glucose | r = 0.05 | 0.66* | No significant correlation between glucose levels and GLS. |
|  | Hemoglobin (Hb) | r = 0.17 | 0.14* | No significant correlation between Hb and GLS. |
|  | Creatinine | r = 0.16 | 0.16* | No significant correlation between creatinine and GLS. |
| **Comorbidities and Complications** | Hypertension | -18.2 ± 1.6 (Yes) / -18.6 ± 1.8 (No) | 0.37** | No significant impact of hypertension on GLS. |
|  | Diabetes Complications | -17.4 ± 1.3 (With) / -18.9 ± 1.6 (Without) | <0.001** | Significantly lower GLS in patients with complications. |
|  | Complication Severity | -18.9 ± 1.7 (None) / -17.5 ± 1.3 (1 Complication) / -16.8 ± 1.3 (≥ 2 Complications) | 0.001*** | GLS decreases with increased complications. |
| **Dyslipidemia and Medication Use** | Dyslipidemia | -18.5 ± 1.7 (With) / -18.3 ± 1.8 (Without) | 0.76** | No significant effect of dyslipidemia on GLS. |
|  | Metformin Use | -18.5 ± 1.8 (Yes) / -18 ± 1.3 (No) | 0.4** | No significant impact of metformin on GLS. |
|  | DPP4-I (Gliptins) | -18.5 ± 1.7 (Yes) / -18.4 ± 1.7 (No) | 0.78** | No significant impact of DPP4-I on GLS. |
|  | Insulin | -18.1 ± 1.5 (Yes) / -18.7 ± 1.9 (No) | 0.16** | No significant impact of insulin on GLS. |
|  | Sulfonylurea | -18.6 ± 1.7 (Yes) / -18.3 ± 1.7 (No) | 0.44** | No significant effect of sulfonylurea on GLS. |
|  | SGLT2-I | -18.4 ± 1.7 (Yes) / -18.4 ± 1.7 (No) | 0.92** | No significant effect of SGLT2-I on GLS. |
|  | Glitazones | -19 (Yes) / -18.4 ± 1.7 (No) | 0.74** | No significant effect of glitazones on GLS. |
|  | GLP1-RA | -19.2 ± 1.2 (Yes) / -18.3 ± 1.7 (No) | 0.12** | No significant effect of GLP1-RA on GLS. |
|  | ACEi/ARB | -18.3 ± 1.8 (Yes) / -18.6 ± 1.7 (No) | 0.44** | No significant effect of ACEi/ARB on GLS. |
|  | Beta-blockers | -18.7 ± 1.4 (Yes) / -18.4 ± 1.8 (No) | 0.58** | No significant impact of beta-blockers on GLS. |
|  | Diuretics | -18.2 ± 1.7 (Yes) / -18.4 ± 1.7 (No) | 0.7** | No significant impact of diuretics on GLS. |
|  | Calcium Channel Blockers | -18.2 ± 1.9 (Yes) / -18.5 ± 1.7 (No) | 0.54** | No significant impact of calcium channel blockers on GLS. |
|  | Aspirin | -18 ± 1.3 (Yes) / -18.5 ± 1.8 (No) | 0.27** | No significant effect of aspirin on GLS. |
|  | Statin | -18.5 ± 1.7 (Yes) / -18.3 ± 1.8 (No) | 0.74** | No significant effect of statins on GLS. |

P-values calculated using: Pearson correlation test (indicated by *), Independent t-test (indicated by **), *One-way ANOVA test (indicated by ***)

**Supplementary Table 2. Correlation Between GLS (%) and echocardiogram parameters in the uncontrolled Diabetes**

| **ECHO Parameter** | **Correlation (r)** | **P-value** | **Interpretation** |
| --- | --- | --- | --- |
| LVEDd | 0.03 | 0.81 | No significant correlation with GLS. |
| **LVESd** | **0.3** | **0.007** | Significant positive correlation with GLS. |
| IVSEDd | -0.5 | 0.68 | No significant correlation with GLS. |
| LVPWd | -0.02 | 0.84 | No significant correlation with GLS. |
| LV Mass | -0.02 | 0.89 | No significant correlation with GLS. |
| LV Mass Index | -0.06 | 0.59 | No significant correlation with GLS. |
| LVEDV | 0.19 | 0.1 | No significant correlation with GLS. |
| **LVESV** | **0.25** | **0.03** | Significant positive correlation with GLS. |
| LA Volume | -0.22 | 0.06 | Trend towards correlation, but not significant with GLS. |
| LVEF | -0.19 | 0.1 | No significant correlation with GLS. |
| E | -0.07 | 0.55 | No significant correlation with GLS. |
| A | -0.04 | 0.76 | No significant correlation with GLS. |
| E/A | -0.02 | 0.86 | No significant correlation with GLS. |
| Mitral Flow | N/A | 0.44 | No significant correlation with GLS. |
| Mitral Deceleration Time | -0.21 | 0.08 | No significant correlation with GLS. |
| Septal e' | -0.05 | 0.69 | No significant correlation with GLS. |
| Lateral e' | 0.12 | 0.31 | No significant correlation with GLS. |
| Lateral E/e' | -0.15 | 0.19 | No significant correlation with GLS. |
| Septal E/e' | 0.1 | 0.38 | No significant correlation with GLS. |

**Comparison of LV Mass Between Uncontrolled Diabetics with and Without Hypertension**

| **Group** | **LV Mass (Mean ± SD)** | **P-value** | **Statistical Test** |
| --- | --- | --- | --- |
| Diabetics with Hypertension | 155.1 ± 40.4 | 0.19 | Independent t-test |
| Diabetics without Hypertension | 139.8 ± 44.3 |  |  |

*Supplementary Figure 1 LV mass between uncontrolled diabetics with hypertension and without hypertension.*

**Intraobserver and interobserver reproducibility of GLS % measurement**

| Variable | Intraobserver | | Interobserver | |
| --- | --- | --- | --- | --- |
|  | ICC | 95% CI | ICC | 95% CI |
| GLS% | 0.99 | 0.97-0.99 | 0.87 | 0.6-0.96 |

Supplementary Figure 2 Interobserver and interobserver reproducibility of GLS % measurement
